# Refractory Thrombocytopenia is the Earliest Diagnostic Criterion for Sinusoidal Obstruction Syndrome in Children

**DOI:** 10.1097/MPH.0000000000002938

**Published:** 2024-08-26

**Authors:** Filippo Consonni, Alice Ciulli, Daniela Cuzzubbo, Stefano Frenos, Maria Chiara Sanvito, Annalisa Tondo, Veronica Tintori, Eleonora Gambineri

**Affiliations:** Departments of * Experimental and Clinical Biomedical Sciences “Mario Serio”; ‡ Health Sciences; § Neurosciences, Psychology, Drug Research and Child Health (NEUROFARBA), University of Florence; † Centre of Excellence, Division of Pediatric Oncology/Hematology, Meyer Children’s Hospital IRCCS, Florence, Italy

**Keywords:** sinusoidal obstruction syndrome, veno-occlusive disease, refractory thrombocytopenia, hematopoietic stem cell transplantation

## Abstract

Sinusoidal obstruction syndrome (SOS) is a life-threatening complication of hematopoietic stem cell transplantation (HSCT), whose diagnostic criteria changed over time to achieve a timelier diagnosis. Recently, pediatric-specific criteria presented by the European Society for Blood and Marrow Transplantation (pEBMT) incorporated transfusion-refractory thrombocytopenia (RT) as an early indicator of SOS in children. However, a comparison of all individual diagnostic parameters belonging to pEBMT and former SOS diagnostic criteria has never been performed. This retrospective study conducted at a pediatric tertiary care hospital analyzed all pediatric HSCT cases diagnosed with SOS among 170 children transplanted from 2009 to 2023. Eleven patients developed SOS during this period (incidence: 11/170, 6.5%). pEBMT, Seattle, and Baltimore criteria were retrospectively applied to the 11 cases and compared, showing that RT was the earliest fulfilled parameter (median onset: 6 d post-HSCT). pEBMT and Seattle criteria identified 11/11 SOS cases, with pEBMT leading to an earlier diagnosis. RT typically manifested before diagnosis, with significantly higher platelet transfusion requirements before diagnosis than after. RT is the earliest satisfied criterion in pediatric SOS and typically occurs in the initial stages of the disease before diagnosis. Further research is needed to identify additional early indicators of pediatric SOS.

Sinusoidal obstruction syndrome (SOS), formerly known as veno-occlusive disease, is a life-threatening complication of hematopoietic stem cell transplantation (HSCT) due to acute damage to hepatic sinusoidal endothelial cells.^1^ The incidence of SOS in pediatric HSCT stands at ∼20%, exceeding that reported in adults.^2^ Clinical manifestations include tender hepatomegaly, ascites, and jaundice, with anicteric presentations being more prevalent in children.^3^ Early diagnosis holds paramount importance, as numerous studies demonstrated improved outcomes with prompt initiation of specific therapies, such as defibrotide.^4–6^

Diagnostic criteria for SOS have evolved over time, with Baltimore^7^ and Seattle^8^ criteria being used irrespective of age during the last decades. These criteria exhibited several limitations, especially in the pediatric population. First, in Baltimore criteria, hyperbilirubinemia is considered a mandatory parameter, thus excluding anicteric SOS cases. Moreover, the time limit of 20 to 21 days after HSCT hindered the diagnosis of late-onset forms. In addition, the 2% to 5% weight gain criterion is often challenging to be precisely assessed in the pediatric population.^9^ In 2018, the pediatric-specific European Society for Blood and Marrow Transplantation (pEBMT) criteria were introduced, addressing the shortcomings of the earlier Baltimore and Seattle criteria while incorporating transfusion-refractory thrombocytopenia (RT) as a new criterion.^2^

Although the association between RT and SOS has been recognized for decades,^8^ its recent inclusion in the pEBMT criteria revealed that it is a particularly useful diagnostic parameter for early detection of pediatric SOS.^10^ RT is defined as a need of ≥1 weight-adjusted platelet substitution/day to maintain institutional transfusion guidelines.^2^ Nevertheless, RT alone must be differentially diagnosed from various transplant-related complications, including transplant-associated thrombotic microangiopathy, infections, and drug-induced thrombocytopenia.^10–13^ Therefore, RT alone is not specific to SOS and is insufficient for an early diagnosis. It is, therefore, paramount to integrate RT with other diagnostic parameters. Moreover, a comparison of all individual diagnostic parameters belonging to Baltimore, Seattle, and pEBMT diagnostic criteria for SOS has never been conducted.

In this study, we present our 15-year experience of SOS from a tertiary care children’s hospital. Results from a retrospective application of all known pediatric SOS diagnostic criteria are presented, with a focus on RT, which confirms to be a parameter displayed at the initial stages of the disease.

## MATERIALS AND METHODS

### Study Population

The study included all patients under 18 years of age who underwent autologous or allogeneic HSCT at the Center of Excellence of Hematology-Oncology of Meyer Children’s Hospital IRCCS (Florence, Italy) between 2009 and 2023. Patients with a diagnosis of SOS, identified using the appropriate International Classification of Diseases (ICD) code, and meeting the diagnostic criteria at the time of transplantation were considered for analysis.

### Data Collection

Clinical records were reviewed to collect relevant demographic, clinical, and laboratory data of the included patients. The data encompassed primary diagnosis, type of transplant, past medical history, conditioning regimen, and various clinical variables. Specific parameters such as height, weight gain, hepatomegaly, bilirubin and creatinine levels, abdomen ultrasound, number and characteristics of platelet transfusions, the need for renal replacement therapy, paracentesis, or respiratory support, encephalopathy, as well as prophylaxis and treatment strategies were recorded.

### Diagnostic Criteria and Grading

The Baltimore,^7^ modified Seattle,^8^ and pEBMT^2^ diagnostic criteria for SOS were retrospectively applied to each patient to determine their fulfillment and to establish the precise day of SOS diagnosis. Day 0 was defined as the day of hematopoietic stem cell infusion. Pediatric EBMT criteria were used to assess grading of the diagnosed SOS.^2^ Resolution of SOS was defined as the first day when the initial SOS diagnostic criteria were no longer met.

### Specific Parameters for SOS Diagnosis and Grading

#### Estimated Glomerular Filtration Rate (eGFR)

eGFR was calculated according to Bedside Schwartz Formula from patient’s age, height, sex, and serum creatinine level.^14^ This parameter provided a measure of renal function, which was crucial to assess SOS grading according to pediatric EBMT criteria.^2^

#### Persistent RT

RT was recently introduced as a new parameter for SOS diagnosis in children by the pEBMT criteria.^2,15^ According to these criteria, RT was defined as a need of ≥11 weight-adjusted platelet substitution/day to maintain institutional transfusion guidelines.^2^

Platelet transfusion requirement (PTR) expressed in mL/kg/d was calculated for each patient both before and after the diagnosis of SOS.^10^

### Ethical Considerations

As this study involved a retrospective analysis of anonymized data from routine clinical practice, no specific informed consent or ethics approval was required, in accordance with institutional policy.

### Statistical Analysis

Statistical analyses were performed using GraphPad Prism (Version 9.0 for Mac, GraphPad Software, San Diego, CA). Metrics data were assessed for normal distribution. Continuous variables were expressed as median and interquartile ranges (IQRs), while categorical variables were presented as numbers (%). For continuous variables, the Student *t* test was used for the comparison of 2 groups displaying a normal distribution, while the Mann-Whitney test was utilized for non-normally distributed data. One-way ANOVA test or Kruskall-Wallis test with multiple comparisons were employed to compare more than 2 groups displaying normal and non-normal distributions, respectively. Categorical variables were analyzed using the χ^2^ test or Fisher exact test, as appropriate. A *P*-value <0.05 was considered statistically significant, indicating a significant difference between groups.

## RESULTS

### Characteristics of Included Patients

From 2009 to 2023, based on ICD coding, 11 patients (median age: 8.8 y; IQR: 6.3 to 12.15; 55.5% females) received a diagnosis of SOS according to diagnostic criteria available at the time of transplantation. During the same period (2009–2023), our center conducted a total of 170 HSCTs, resulting in an estimated incidence of SOS of 11/170 (6.5%). The baseline characteristics of included patients and main risk factors for SOS are summarized in Table 1. Nine of 11 (81.8%) underwent allogenic and 2/11 (18.2%) had autologous HSCT. 7/11 (63.6%) were affected by a malignant disorder and were previously exposed to chemotherapy. Two of 11 (18.2%) had thalassemia major and 2/11 (18.2%) were affected by rare inborn errors of immunity (prolidase deficiency and STAT3 gain-of-function) not associated with a defect in DNA repair and hence not prone to develop toxic complications of HSCT.^16–18^ Three of 11 (27.3%) were previously treated with IO, while for 1 patient (9.1%) it was the second HSCT. The most prevalent type of conditioning was Busulfan-based (5/11, 45.5%).

**TABLE 1:** Patient and Transplant Characteristics of the Included Cohort

Patient and transplant characteristics	n=11
Sex, n (%)	
Female	6 (54.5)
Male	5 (45.5)
Age at transplantation (y), median (IQR)	8.8 (6.3-12.1)
Disease at transplantation, n (%)	
B-ALL	5 (45.5)
Inborn error of immunity	2 (18.2)
Thalassemia major	2 (18.2)
Neuroblastoma	1 (9.1)
Ewing Sarcoma	1 (9.1)
Donor type, n (%)	
Autologous	2 (18.2)
MSD	1 (9.1)
MUD	1 (9.1)
MMUD	2 (18.2)
Haploidentical	5 (45.5)
Stem cell source, n (%)	
Peripheral blood	4 (36.45)
Bone marrow	5 (45.5)
Conditioning regimen, n (%)	
MAC	11 (100)
Busulfan-based	5 (45.5)
Treosulfan-based	2 (18.2)
TBI-based	4 (36.6)
Previous exposure to chemotherapy, n (%)	7 (63.7)
Previous exposure to IO, n (%)	3 (27.3)
Prophylactic treatment, n (%)	
Ursodeoxycholic acid	2 (18.2)
Defibrotide	2 (18.2)

B-ALL indicates B-cell acute lymphoblastic leukemia; IO, Inotuzumab Ozogamicin; IQR, interquartile range; MAC, myeloablative conditioning; MMUD, mismatched unrelated donor; MSD, matched sibling donor; MUD, matched unrelated donor; TBI, total body irradiation.

### Diagnosis of SOS and Retrospective Application of Diagnostic Criteria

**FIGURE 1:**
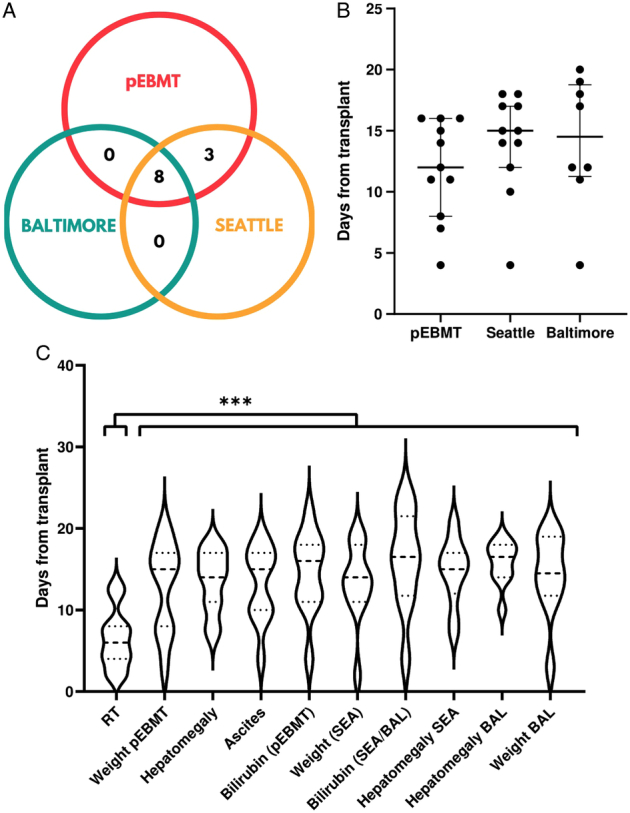
Retrospective assessment of SOS diagnostic criteria in included patients. A, Venn diagram showing the number of patients diagnosed through a retrospective application of each diagnostic criteria. B, Scatter plot showing the time required for each criterion to be satisfied in included patients. C, Violin plot showing the time required for each specific parameter of the 3 assessed criteria to be satisfied. Refractory thrombocytopenia (RT) was satisfied significantly earlier than any other parameter. BAL/Baltimore indicates Baltimore criteria; pEBMT, pediatric European Society for Blood and Marrow Transplantation criteria; SEA/Seattle, modified Seattle criteria. ***P<0.001.

The median time from HSCT to SOS diagnosis was 14 days (IQR: 9.5 to 15.5). At the time of diagnosis, the majority (6/11, 54.5%) of SOS cases were detected through pEBMT criteria, 5/11 (45.5%) using Seattle criteria, while no patients were diagnosed with Baltimore criteria (Table 2). Retrospective application of the 3 diagnostic criteria showed that pEBMT and Seattle identified 11/11 (100%) of cases, while Baltimore criteria were satisfied in 8/11 (72.7%) included patients (Fig. 1A). Median time to diagnosis was shorter with pEBMT criteria (12 d, IQR: 9.5 to 15.5), than with Seattle (15 d, IQR: 13 to 17) and Baltimore criteria (16.5 d, 12 to 18.3) (Fig. 1B). One patient (9.1%) displayed anicteric SOS, while no late-onset presentations were detected.

**TABLE 2:** Detailed Characteristics of Included Patients

Patient	Age at HSCT (yr)	Year of HSCT	Diagnosis	Donor type	Stem cell source	Conditioning	Previous IO	Defibrotide treatment (start day)	Total days of defibrotide	pEBMT (d)	Modified Seattle (d)	Baltimore (d)	LSM: basal; first positive (d)	Grading	Days needed to SOS resolution	Outcome
P1	11.6	2023	2nd relapse, refractory B-ALL	MUD	PBSC	TBI-FLU	Yes (-5 mo)	(+11)	26	11*	17	17	4.3 kPa; 25.4 kPa (+13)	Severe	23	Alive and well
P2	15.6	2022	3rd relapse (after CAR-T) B-ALL	Haplo	PBSC	TBI-FLU (+PT-CY)	Yes (-1.5 mo)	Prophylaxis (-7), unmodified dose after diagnosis	52	7*	14	12	7.1 kPa; 8.8 kPa (+1)	Severe	29	Alive and well
P3	4.2	2021	Thalassemia major	Haplo	BM	BU-FLU (+PT-CY)	No	(+10)	24	8*	10	11		Severe	16	Alive and well
P4	4.6	2021	Thalassemia major	Haplo	BM	BU-FLU (+PT-CY)	No	(+17)	22	14*	15			Severe	19	Alive and well
P5	8.9	2019	1st relapse B-ALL	Haplo	PBSC	TBI-FLU-TT (+PT-CY)	No	(+17)	28	16*	17	18		Severe	20	Alive and well
P6	1.1	2014	Neuroblastoma	Auto	PBSC	BU-MEL	No	(+15)	24	15	15*			Severe	16	Alive and well
P7	8.2	2017	3rd relapse B-ALL (2nd HSCT, previous TBI), after CAR-T	Haplo	PBSC	TT-TREO-FLU (+PT-CY)	Yes (-20 d)	(+3)	28	4	4*	16		Very severe	N.A. (death)	Died of MOF caused by SOS
P8	12.7	2018	B-ALL	MMUD	BM	TBI-FLU	No	(+12)	24	11*	12	12		Severe	14	B-ALL progression (2 years later)
P9	8.8	2016	IEI (STAT3-GoF)	MUD	BM	TT-TREO-FLU	No	(+18)	22	12	14*			Severe	13	Alive and well
P10	15.1	2010	Ewing sarcoma	Auto	PBSC	BU-MEL	No	(+24) SOS relapse	45	16	18*	19		Severe	82 (SOS relapse)	Alive and well
P11	8	2009	IEI (prolidase deficiency)	MSD	BM	BU-CY	No	Prophylaxis (-7), unmodified dose after diagnosis	30	16	18*	20		Very severe	N.A. (death)	Died of MOF caused by SOS

Days are intended as days from stem cells infusion (day 0).

* Diagnostic criteria were employed to make the diagnosis of SOS at the time of HSCT. Baltimore and Modified Seattle criteria were first described in 1987 and 1993, respectively, while pEBMT criteria were introduced more recently (2018).

B-ALL indicates B-cell acute lymphoblastic leukemia; BM, bone marrow; BU, busulfan; CAR-T, chimeric antigen receptor T cells; FLU, fludarabine; GoF, gain-of-function; Haplo, haploidentical; HSCT, hematopoietic stem cell transplantation; IEI, inborn error of immunity; IO, Inotuzumab Ozogamicin; LSM, liver stiffness measurement; MEL, melphalan; MMUD, mismatched unrelated donor; Auto, autologous; MOF, multiorgan failure; MSD, matched sibling donor; MUD, matched unrelated donor; NA, not applicable; PBSC, peripheral blood stem cells; pEBMT, pediatric European Society for Blood and Marrow Transplantation criteria; PT-CY, post-transplant cyclophosphamide; SOS, sinusoidal obstruction syndrome; TBI, total body irradiation; TREO, treosulfan; TT, thiotepa.

### Early Apparition of Refractory Thrombocytopenia and Late Development of Hyperbilirubinemia

**FIGURE 2:**
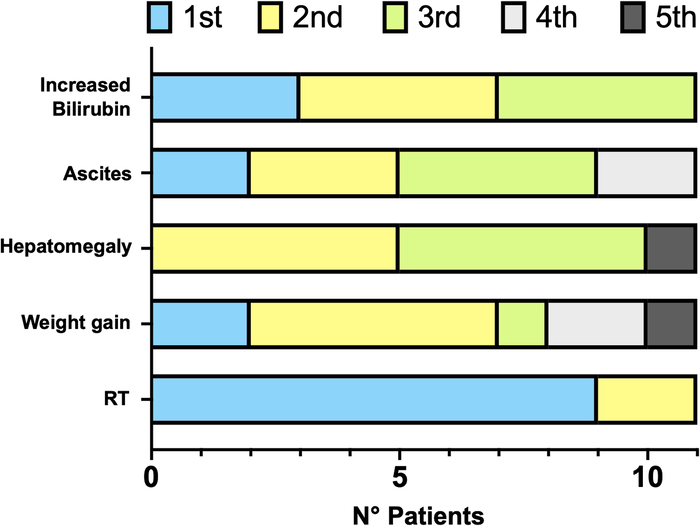
Order of appearance of the 5 parameters belonging to pEBMT criteria in included patients. pEBMT indicates pediatric European Society for Blood and Marrow Transplantation criteria; RT, refractory thrombocytopenia.

Comparison of all parameters of Seattle, Baltimore and pEBMT criteria showed that time from HSCT to the apparition of RT (median: 6 d; IQR: 4 to 8) was significantly shorter than time to satisfy any other parameters (*P*=0.0005) (Fig. 1C). Considering only the 5 parameters included in pEBMT criteria, RT was the first SOS manifestation in 9/11 (81.8%) subjects (Fig. 2) and appeared significantly earlier than the other 4 parameters (*P*=0.001).

**FIGURE 3:**
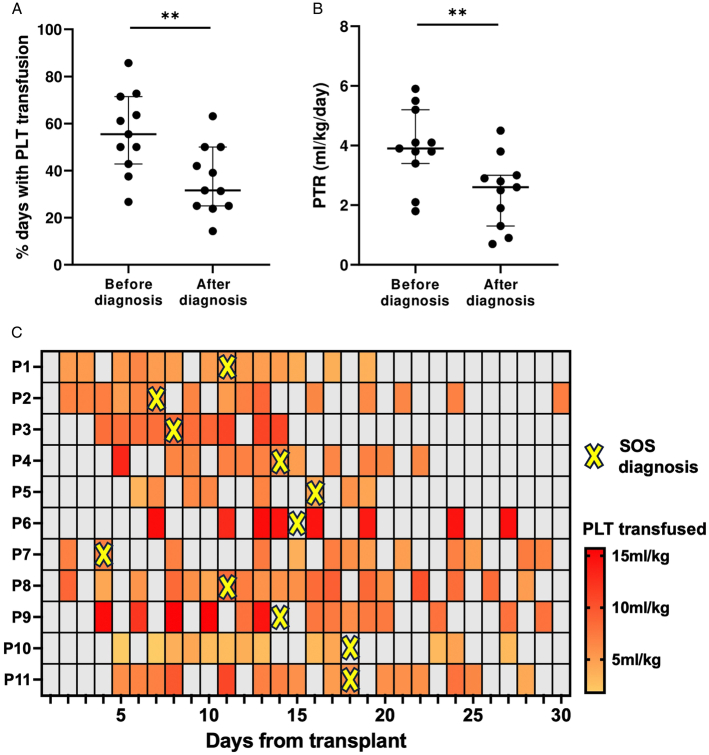
Refractoriness to platelet transfusions before and after diagnosis of SOS in included patients. A, Percentage of days in which the patient required platelet (PLT) transfusions before and after diagnosis of SOS. B, Platelet transfusion requirement (PTR) before and after diagnosis of SOS. C, Heat map showing the days when platelets were transfused (colored squares), and the amount (mL/kg) of platelet transfused for each included patient. Gray squares indicate days where platelets were not transfused. The yellow cross indicates the day when the diagnosis of SOS was established in clinical practice. The heat map shows that the days where platelets were transfused are highly distributed in the prediagnosis period.

Before diagnosis, platelet transfusions were required in a median of 55.5% of days (IQR: 48.2 to 67.5) with a median platelet transfusion requirement (PTR) of 3.9 mL/kg/d (IQR: 3.6 to 4.6). After diagnosis, the median percentage of days requiring platelet transfusions and PTR were 31.6% (IQR: 25 to 46.1) and 2.6 mL/kg/d (IQR: 1.6 to 2.9), respectively. The differences between prediagnosis and postdiagnosis periods were statistically significant (*P*=0.0073 and 0.0091, respectively) (Figs. 3A–C).

Moreover, 9/11 (81.8%) patients were anicteric at diagnosis, and among them 8/9 (88.9%) later developed hyperbilirubinemia. Peak hyperbilirubinemia (median: 3.8 mg/dl; IQR: 3.1 to 7) was reached at a median time of 9 days (IQR: 5 to 12) after diagnosis (Table, Supplemental Digital Content 1, http://links.lww.com/JPHO/A702).

### Severity Grading and Outcome

Application of pEBMT severity grading^2^ revealed that 9/11 (81.8%) included patients had severe SOS, while 2/11 (18.2%) were very severe cases. Seven of 11 patients (63.6%) required paracentesis, 2/11 (18.2%) needed continuous positive airway pressure (CPAP) ventilation, 9/11 (81.8%) had replacement of coagulation factors, while none required renal replacement therapy. Two of 11 (18.2%) died of multiorgan failure due to very severe SOS. One patient (P8) died during follow-up (2 y after SOS) for disease progression. Resolution of SOS in survived patients occurred after a median time of 19 days (IQR: 16 to 23) from diagnosis. One patient (P10) presented a relapse of SOS after defibrotide suspension and was therefore treated a second time with final resolution of SOS manifestations at day +82 after HSCT (Table, Supplemental Digital 2, http://links.lww.com/JPHO/A703).

### Prophylaxis and Treatment

All included patients received defibrotide: in 2/11 (18.2%) cases it was administered as prophylaxis, while 9/11 (81.8%) received it upon diagnosis. Similarly, 2/11 (18.2%) and 9/11 (81.8%) patients received prophylactic and therapeutic ursodeoxycholic acid, respectively. One of 11 (9.1%) received steroids, and all patients needed diuretics as supportive treatment (Table, Supplemental Digital 3, http://links.lww.com/JPHO/A704). The median time from diagnosis (with clinical criteria available at the time) to the start of defibrotide was 1 day (IQR: 1 to 3), while the mean duration of defibrotide treatment was 26 days (IQR: 24 to 29).

## DISCUSSION

Early diagnosis of SOS is paramount to promptly initiate specific treatments such as defibrotide. For this reason, diagnostic criteria for SOS evolved over time, and since 2018, specific criteria for children have been introduced to increase the likelihood of a timely SOS diagnosis.^2^ A recent retrospective analysis of a large cohort at a single American hospital was conducted by Ragoonanan et al,^19^ demonstrating that the pEBMT criteria are more sensitive compared with the previous ones. However, a direct comparison of each individual parameter of the 3 diagnostic criteria has never been reported.

In this study, we performed a similar retrospective analysis with the additional aim of evaluating why the pEBMT criteria are the most sensitive. Both pEBMT and Seattle criteria managed to detect all cases of SOS, but the former allowed an earlier diagnosis. Conversely, the application of the Baltimore criteria missed 3/11 (27.3%) cases, which is consistent with previous estimates.^2^ The increased sensitivity and earliness of pEBMT criteria are primarily due to the RT parameter, which in our cohort was found in 100% of patients, with a median time of onset of 6 days after HSCT. These findings are in line with those reported by Embaby et al,^10^ who first confirmed that RT is a valid early indicator of SOS in children. This study further adds that RT, when compared with each parameter in all other diagnostic criteria, is satisfied significantly earlier.

Furthermore, this study demonstrated for the first time that RT mainly occurs before SOS diagnosis, since both the number of platelet transfusions and the PTR were significantly higher before diagnosis than after. The reduction of the need for platelet transfusions following diagnosis could be attributed both to the improvement of the SOS tableau upon introduction of defibrotide and to engraftment, that contributes to increasing the circulating pool of platelets. Moreover, similarly to previous studies,^19^ hyperbilirubinemia was confirmed to be a late sign of SOS, since most included patients were anicteric at diagnosis, but later developed jaundice with peak bilirubin levels occurring at an average of 9 days after diagnosis. The only anicteric case of SOS in our cohort was diagnosed using pEBMT criteria, further confirming their sensitivity in detecting such cases.^20^

Most cases in this cohort were severe or very severe SOS, similarly to what was described in a large retrospective survey on Italian children,^21^ while the incidence of SOS reported in this study (6.5%) is significantly lower than that described in other pediatric cohorts.^2,22,23^ It is possible, therefore, that some diagnoses of mild/moderate SOS have been missed over the years. However, an extensive retrospective application of SOS diagnostic criteria to recalculate the incidence of SOS in our HSCT Unit goes beyond the goals of this study and is not feasible due to lack of data. Importantly, the patient recruitment period for this study predates the introduction of pEBMT criteria. As suggested by recent data,^22^ it is possible that pEBMT criteria increased the diagnosis of mild-moderate SOS, which might explain the skew toward severe cases in our cohort, as some patients were diagnosed before the pEBMT criteria were introduced.^2^

The main limitations of this study are due to its single-center and retrospective nature. The small sample size reduces the statistical power of the analyses, and the retrospective nature results in a lack of data, preventing recalculation of SOS incidence in the Unit using pEBMT criteria. Future investigations should explore additional early indicators of SOS in children, which, in combination with RT, could facilitate early diagnosis and timely initiation of appropriate treatment. Initial reports on liver stiffness measurement by transient elastography (TE) appear promising for this purpose.^24,25^ An ongoing prospective study (ElastoVOD trial, NCT03426358 at clinicaltrials.gov) is expected to provide further insights into the role of TE in SOS and its potential association with RT in the early diagnosis of SOS.

In conclusion, we retrospectively applied all known diagnostic criteria for SOS (pEBMT, Seattle, and Baltimore) to all cases diagnosed at our center, revealing that RT is the earliest single criterion to be satisfied in children. Moreover, we demonstrated that RT typically occurs in the initial stages of the disease, before diagnosis. Further research is needed to identify additional early indicators of SOS that can enhance the specificity of RT in the diagnosis of this life-threatening complication of HSCT.

## Supplementary Material

SUPPLEMENTARY MATERIAL
