# Supplementary material for: Refractory Thrombocytopenia is the Earliest Diagnostic Criterion for Sinusoidal Obstruction Syndrome in Children
Source: J Pediatr Hematol Oncol. 2024 Aug 26;46(7):e501–7. doi: 10.1097/MPH.0000000000002938 (PMC11426974; doi:10.1097/MPH.0000000000002938)
Supplement: SUPPLEMENTARY MATERIAL [file mph-46-e501-s001.docx]

**Supplemental Digital Content 1.** Total bilirubin values in included patients throughout the transplant period.

| **Patient** | **SOS diagnosis (day)** | **Total bilirubin at diagnosis (mg/dl)** | **Peak total bilirubin (mg/dl)** | **Day of bilirubin peak** | **Δ day diagnosis-peak bilirubin** |
| --- | --- | --- | --- | --- | --- |
| P1 | 11 | 0.8 | 3.8 | 16 | 5 |
| P2 | 7 | 0.9 | 3.4 | 22 | 15 |
| P3 | 8 | 0.6 | 4.1 | 13 | 5 |
| P4 | 14 | 0.6 | 3.1 | 26 | 12 |
| P5 | 16 | 1.8 | 6.7 | 21 | 5 |
| P6 | 15 | 0.4 | 2.4 | 24 | 9 |
| P7 | 4 | 2.8 | 13.8 | 34 | 30 |
| P8 | 11 | 1 | 7.3 | 20 | 9 |
| P9 | 14 | 0.7 | 1.8 | 18 | 4 |
| P10 | 18 | 1.2 | 3.1 | 30 | 12 |
| P11 | 18 | 3.4 | 21 | 30 | 12 |
| **Median (IQR)** | **14 (9.5-15.5)** | **0.9 (0.6-1.5)** | **3.8 (3.1-7)** | **22 (19-18)** | **9 (5-12)** |

SOS; sinusoidal obstruction syndrome; Δ, difference; IQR, interquartile range.
