# Supplementary material for: Refractory Thrombocytopenia is the Earliest Diagnostic Criterion for Sinusoidal Obstruction Syndrome in Children
Source: J Pediatr Hematol Oncol. 2024 Aug 26;46(7):e501–7. doi: 10.1097/MPH.0000000000002938 (PMC11426974; doi:10.1097/MPH.0000000000002938)
Supplement: SUPPLEMENTARY MATERIAL [file mph-46-e501-s002.docx]

**Supplemental Digital Content 2.** Grading parameters of included patients according to EBMT severity grading.

| **Grading parameters** | **n = 11** |
| --- | --- |
| **AST/ALT increase**  >2x and <5x  > 5x | 1 (9.1%)  10 (91%) |
| **Persistent RT**  3-7 days  > 7 days | 3 (27.3%)  8 (72.7%) |
| **Bilirubin > 2 mg/dl** | 10 (91%) |
| **Ascites**  Moderate  Necessity for paracentesis | 4 (36.4%)  7 (63.7%) |
| **Bilirubin doubling within 48h** | 7 (63.7%) |
| **Coagulation**  Impaired  Impaired with need for replacement of CF | 10 (91%)  9 (81.8%) |
| **Renal function (eGFR)**  Normal  Mildly altered  Moderate | 7 (63.7%)  2 (18.2%)  2 (18.2%) |
| **Pulmonary function (O_2_ requirement)**  < 2 L/min  > 2 L/min  HFNC | 2 (18.2%)  3 (27.3%)  5 (45.5%) |
| **CNS: new onset cognitive impairment** | 2 (18.2%) |

AST, aspartate transaminase; ALT, alanine aminotransferase; RT, refractory thrombocytopenia; eGFR, estimated glomerular filtration rate; O2, oxygen; HFNC, high flow nasal cannula; CNS, central nervous system.
