# Supplementary material for: Refractory Thrombocytopenia is the Earliest Diagnostic Criterion for Sinusoidal Obstruction Syndrome in Children
Source: J Pediatr Hematol Oncol. 2024 Aug 26;46(7):e501–7. doi: 10.1097/MPH.0000000000002938 (PMC11426974; doi:10.1097/MPH.0000000000002938)
Supplement: SUPPLEMENTARY MATERIAL [file mph-46-e501-s003.docx]

**Supplemental Digital Content 3.** Administered treatment in included patients

| **Patient** | **Diuretics** | **Defibrotide prophylaxis (day)** | **Defibrotide therapy (day)** | **Ursodeoxycholic acid (day)** | **Steroids** | **Paracentesis** |
| --- | --- | --- | --- | --- | --- | --- |
| P1 | Yes | No | Yes (+11) | Yes, prophylaxis | No | No |
| P2 | Yes | Yes (-7) | Yes, prophylaxis | Yes, prophylaxis | No | Yes |
| P3 | Yes | No | Yes (+10) | Yes (+12) | No | Yes |
| P4 | Yes | No | Yes (+17) | Yes (+17) | No | No |
| P5 | Yes | No | Yes (+17) | No | No | Yes |
| P6 | Yes | No | Yes (+15) | Yes (+18) | No | Yes |
| P7 | Yes | No | Yes (+3) | No | No | Yes |
| P8 | Yes | No | Yes (+12) | Yes (+14) | No | Yes |
| P9 | Yes | No | Yes (+18) | Yes (+18) | No | No |
| P10 | Yes | No | Yes (+24) | Yes (+21) | No | Yes |
| P11 | Yes | Yes (-7) | Yes, prophylaxis | Yes (+19) | Yes | No |
| **Total, no. (%)** | **11 (100%)** | **2 (18.2%)** | **11 (100%)** | **9 (81.8%)** | **1 (9.1%)** | **7 (63.3%)** |

Days are intended as days from stem cells infusion (day 0). no., number.
